# Supplementary material for: Global critical soil moisture thresholds of plant water stress
Source: Nat Commun. 2024 Jun 6;15:4826. doi: 10.1038/s41467-024-49244-7 (PMC11156669; doi:10.1038/s41467-024-49244-7)
Supplement: Supplementary file 1 — Supplementary Information [file 41467_2024_49244_MOESM1_ESM.pdf]

Supplementary Information

Global critical soil moisture thresholds of plant water stress

Zheng Fu[1, 2*], Philippe Ciais[2], Jean-Pierre Wigneron[3], Pierre Gentine[4], Andrew F. Feldman[5, 6], David Makowski[7], Nicolas Viovy[2], Armen R. Kemanian[8], Daniel S. Goll[2], Paul C. Stoy[9], I. Colin Prentice[10, 11], Dan Yakir[12], Liyang Liu[2], Hongliang Ma[13], Xiaojun Li[3], Yuanyuan Huang[1], Kailiang Yu[2], Peng Zhu[14], Xing Li[15], Zaichun Zhu[16], Jinghui Lian[2], William K. Smith[17]

[1] Key Laboratory of Ecosystem Network Observation and Modeling, Institute of Geographic Sciences and Natural Resources Research, Chinese Academy of Sciences, Beijing, 100101, China
[2] Laboratoire des Sciences du Climat et de l'Environnement, LSCE/IPSL, CEA-CNRS-UVSQ, Université Paris-Saclay, Gif-sur-Yvette, 91191, France
[3] ISPA, INRAE, Université de Bordeaux, Bordeaux Sciences Agro, F-33140, Villenave d'Ornon, France
[4] Department of Earth and Environmental Engineering, Columbia University, New York, NY, 10027, USA
[5] NASA Goddard Space Flight Center, Biospheric Sciences Laboratory, Greenbelt, MD 20771, USA
[6] Earth System Science Interdisciplinary Center, University of Maryland, College Park, MD, USA
[7] Unit Applied mathematics and computer science (UMR MIA-PS) INRAE AgroParisTech Université Paris-Saclay, Palaiseau, 91120 France
[8] Department of Plant Science, The Pennsylvania State University, 116 Agricultural Science and Industries Building, University Park, PA 16802, USA
[9] Department of Biological Systems Engineering, University of Wisconsin – Madison, USA
[10] Georgina Mace Centre for the Living Planet, Department of Life Sciences, Imperial College London, Silwood Park Campus, Buckhurst Road, Ascot, SL5 7PY, UK
[11] Ministry of Education Key Laboratory for Earth System Modeling, Department of Earth System Science, Tsinghua University, Beijing 100084, China
[12] Earth and Planetary Sciences, Weizmann Institute of Science, Rehovot 7610001, Israel
[13] INRAE, Avignon Universit´e, UMR 1114 EMMAH, UMT CAPTE, F-84000, Avignon, France
[14] Department of Geography, The University of Hong Kong, Hong Kong SAR, China
[15] Research Institute of Agriculture and Life Sciences, Seoul National University, Seoul, South Korea
[16] Peking University Shenzhen Graduate School, Peking University, Shenzhen 518055, Guangdong, China
[17] School of Natural Resources and the Environment, University of Arizona, Tucson, AZ, USA

[*]Correspondence author: fuzheng@igsnrr.ac.cn

Contents of this file

Supplementary Figures 1 to 12
Supplementary Tables 1 to 3

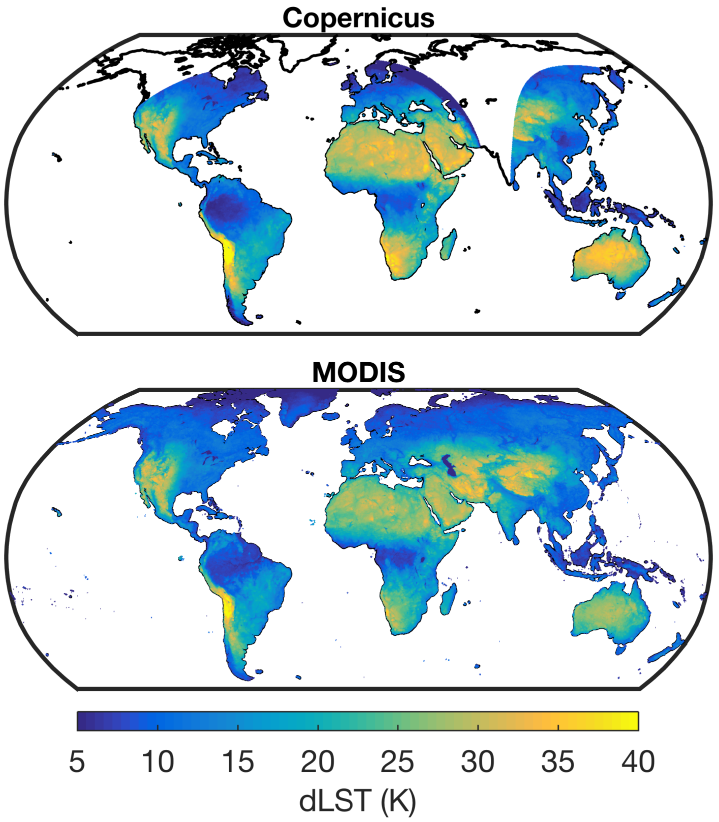

Supplementary Fig. 1. Global distribution of land surface temperature diurnal amplitude (dLST) using Copernicus and MODIS land surface temperature (LST) data. An example showed the median dLST in 2020 using Copernicus and MODIS LST data, respectively. Note that there is no geostationary satellite coverage in parts of northern and eastern Europe, Central Asia, and the Indian subcontinent as well as parts of eastern Siberia and northern North America for Copernicus LST.

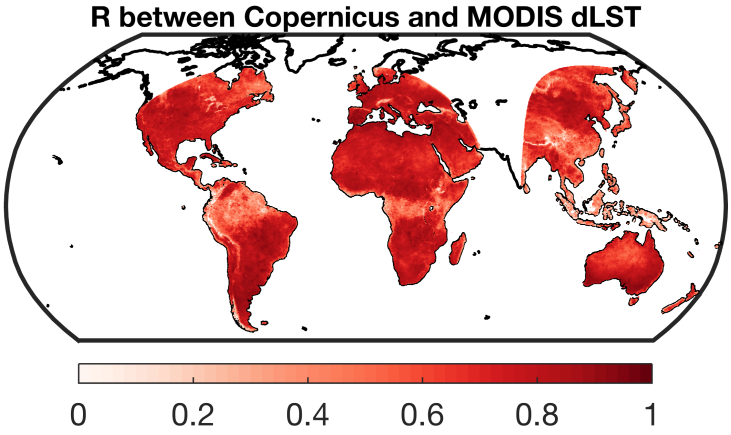

Supplementary Fig. 2. Global distribution of the correlation coefficient between Copernicus and MODIS land surface temperature diurnal amplitude (dLST). An example showed the correlation coefficient (R) between Copernicus and MODIS daily dLST in 2020. Note that there is no geostationary satellite coverage in parts of northern and eastern Europe, Central Asia, and the Indian subcontinent as well as parts of eastern Siberia and northern North America for Copernicus LST.

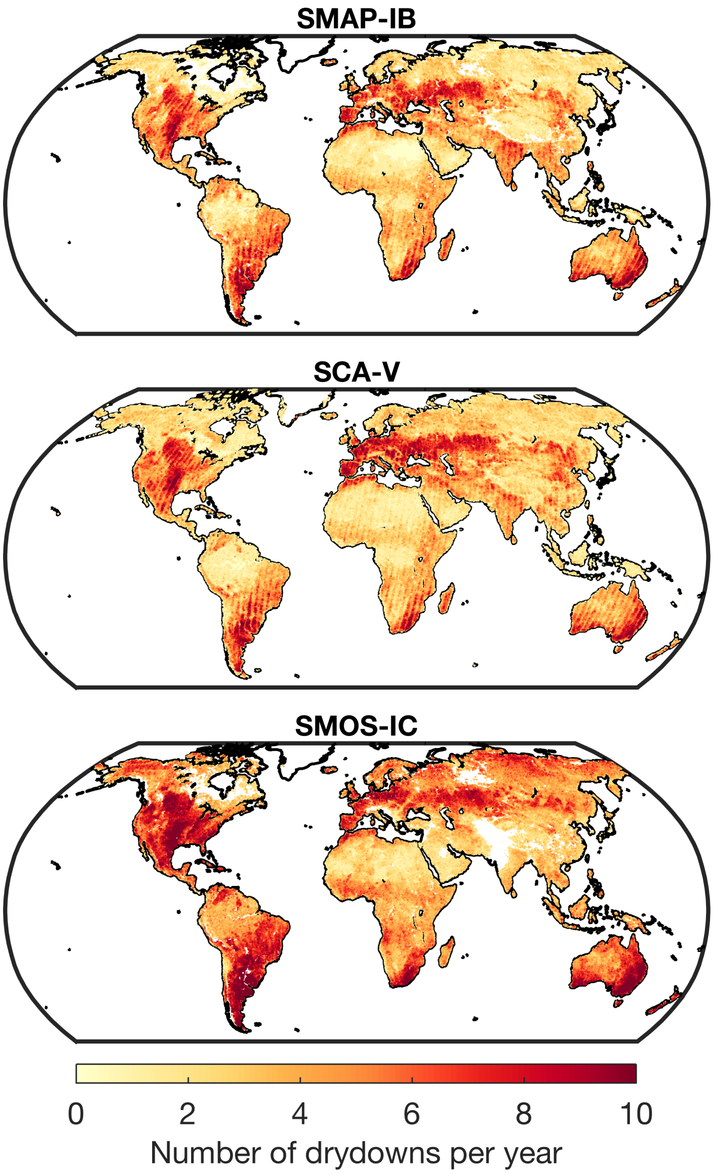

Supplementary Fig. 3. Global distribution of the number of soil dry-downs per year. The median number of soil dry-downs per year during 2016-2020 calculated from SMAP-IB, SCA-V and SMOS-IC, respectively.

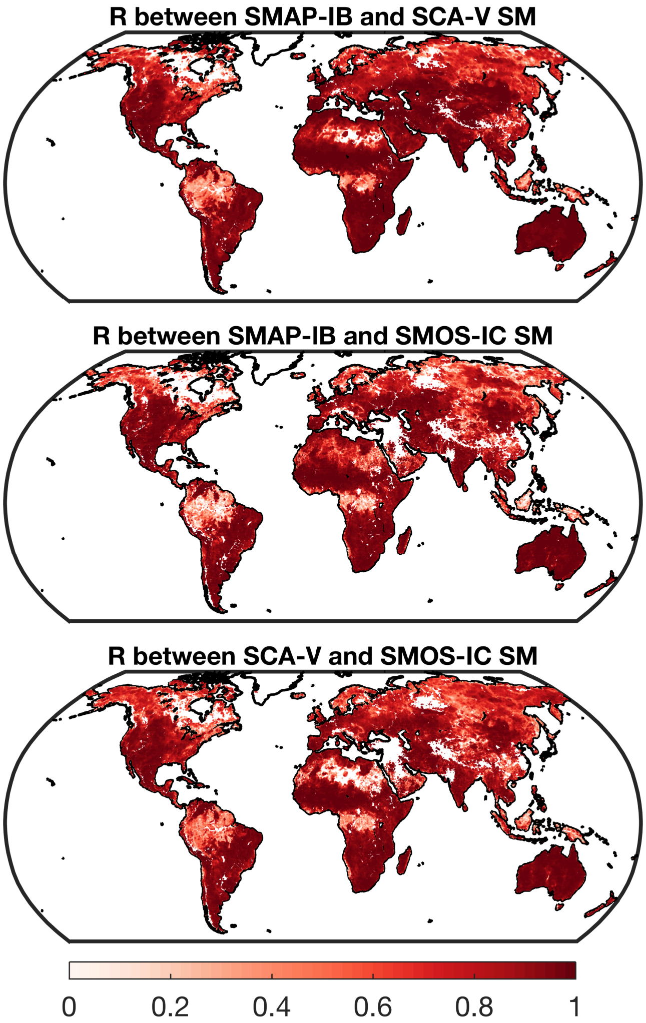

Supplementary Fig. 4. Global distribution of the correlation coefficient among SMAP-IB, SCA-V and SMOS-IC daily soil moisture (SM). An example showed the correlation coefficient (R) among SMAP-IB, SCA-V and SMOS-IC daily SM in 2020.

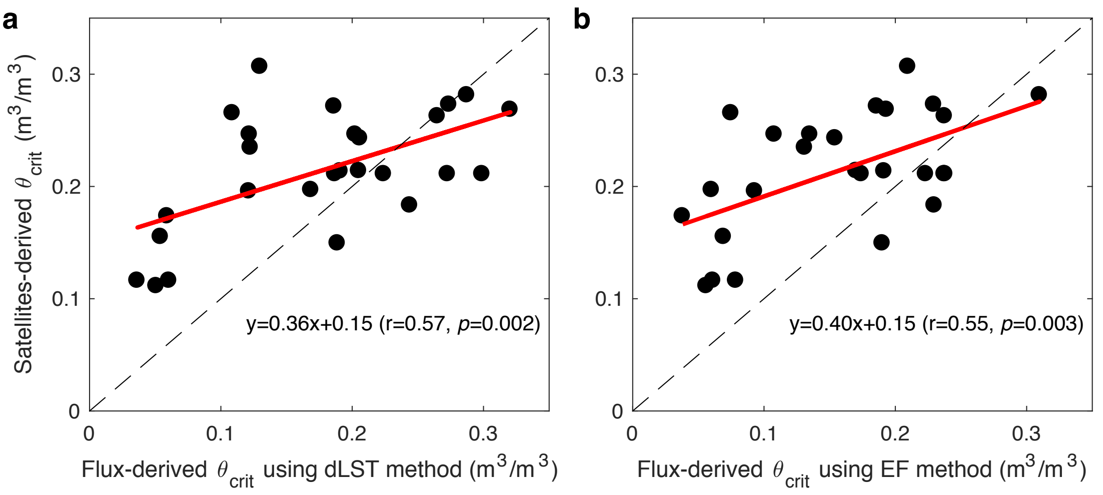

Supplementary Fig. 5. Comparison of critical soil moisture thresholds (θ_{crit}) based on satellite observations and flux towers across sites. Relationships between θ_{crit} derived from satellite ensembles and θ_{crit} derived from flux towers using the land surface temperature diurnal amplitude (dLST)–SM method (**a**) and the evaporative fraction (EF)–SM method (**b**). The red line is the linear regression line while the dashed line represents the 1:1 line.

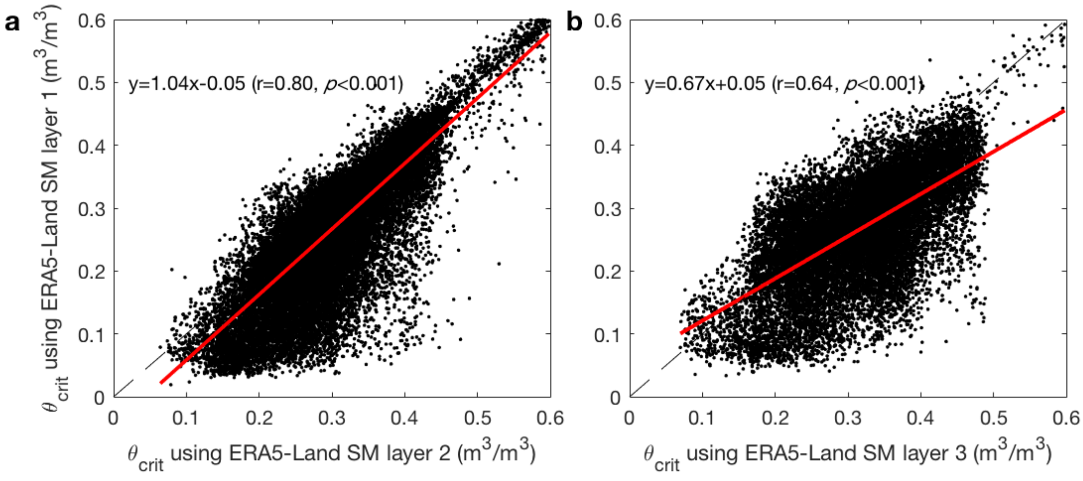

Supplementary Fig. 6. Comparison of critical soil moisture thresholds (θ_{crit}) estimated from ERA5-Land soil moisture (SM) between different soil layers. Comparison of θ_{crit} estimated from ERA5-Land SM layer 1 (0-7 cm depth) with the layers 2 (7-28 cm, **a**) or 3 (28-100 cm, **b**). SM estimates from deeper layers (layers 4 and 5) in ERA5-Land are less constrained by observations, so we did not use them. The red line is the linear regression line while the dashed line represents the 1:1 line.

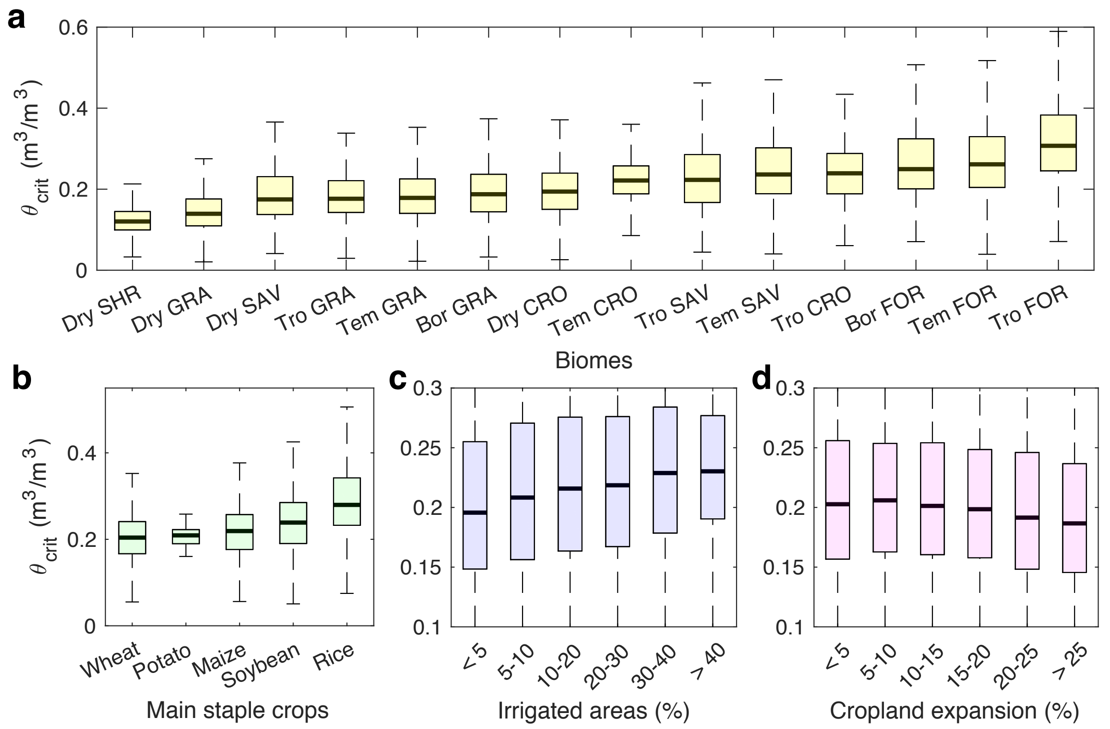

Supplementary Fig. 7. The critical soil moisture thresholds (θ_{crit}) among different biomes and the impacts of crop species, irrigated areas and cropland expansion on θ_{crit}. a, The θ_{crit} among different biomes. **b-d,** The impacts of crop species, irrigated areas and cropland expansion on θ_{crit} of crops. For each box plot, the middle line indicates the median; the box indicates the upper and lower quartiles, and the whiskers indicate the 5th and 95th percentiles of the data. The geographic distribution of main staple crops was from Monfreda, Ramankutty [1]. Global Map of Irrigation Areas was downloaded from The Food and Agriculture Organization[2], showing the amount of area equipped for irrigation in percentage of the total area on a raster. For cropland expansion, the map of percent of cropland net gain per pixel during 2003-2019 was from Potapov, Turubanova [3]. SHR: shrublands; GRA: grasslands; SAV: savannas; CRO: croplands; FOR: forests; Tro: tropical; Tem: temperate; Bor: boreal.

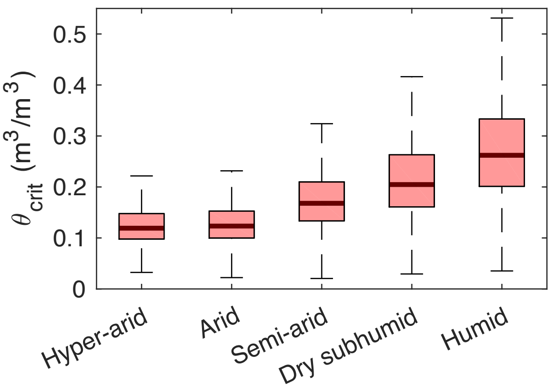

Supplementary Fig. 8. The critical soil moisture thresholds (θ_{crit}) among different climate types based on the aridity classification by the United Nations Environment Programme[4]. For each box plot, the middle line indicates the median; the box indicates the upper and lower quartiles, and the whiskers indicate the 5th and 95th percentiles of the data.

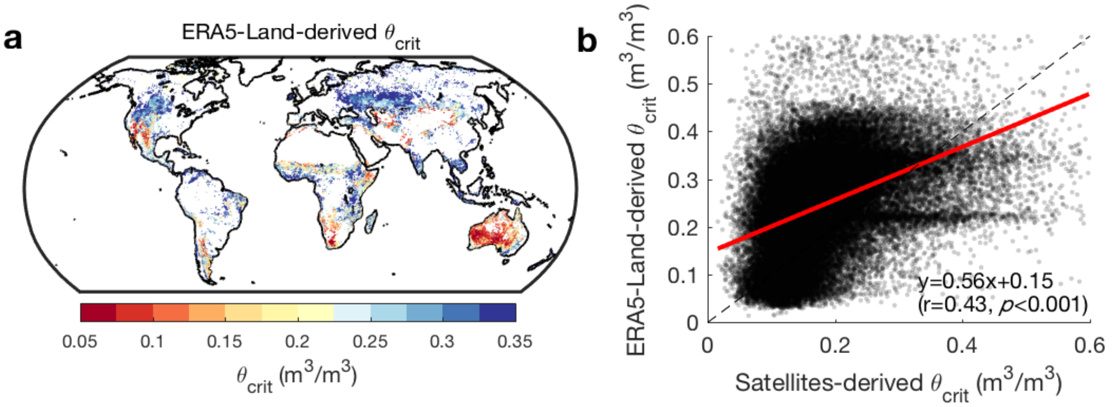

Supplementary Fig. 9. The global distribution of critical soil moisture threshold (θ_{crit}) using ERA5-Land data and the relationship between θ_{crit} derived from ERA5-Land and θ_{crit} derived from satellite ensembles. a, The global distribution of θ_{crit} using ERA5-Land surface soil moisture (SM) and land surface temperature diurnal amplitude (dLST). The gaps indicate that the pixels did not have a defined θ_{crit} value because there were either no dry-downs, or SM varied only within a water- or energy-limited regime, or the number of samples were too low (missing data), thus rendering the breakpoint analysis of dLST–SM unreliable. **b**, The relationship between θ_{crit} derived from ERA5-Land and θ_{crit} derived from satellite ensembles. The red line is the linear regression line while the dashed line represents the 1:1 line.

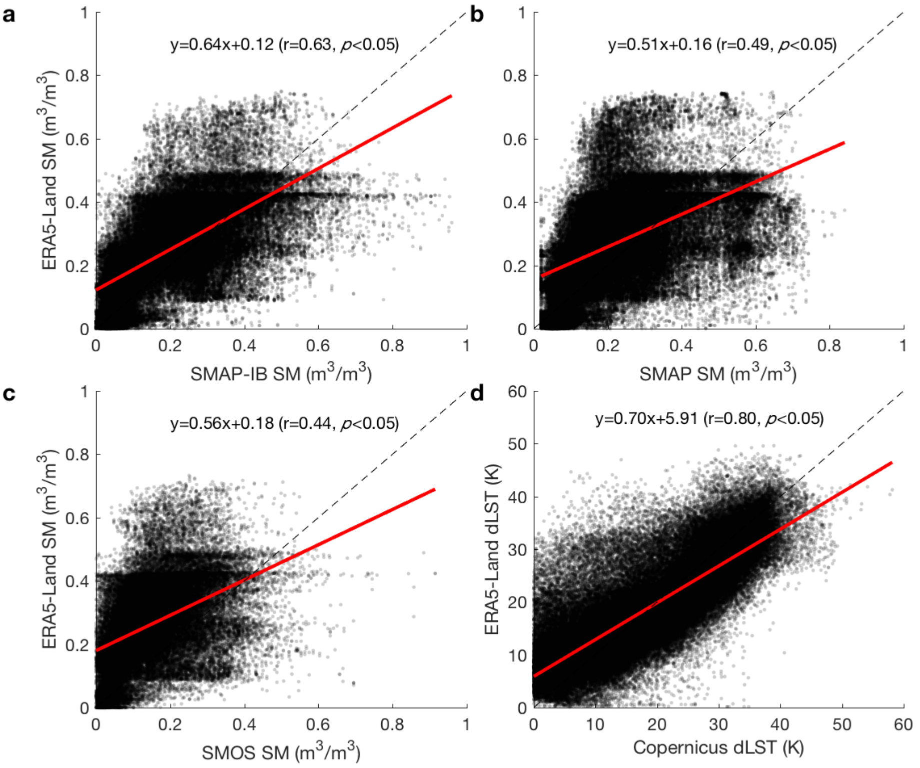

Supplementary Fig. 10. Comparison of daily ERA5-Land soil moisture (SM) and land surface temperature diurnal amplitude (dLST) with those from satellites for a day in 2020.

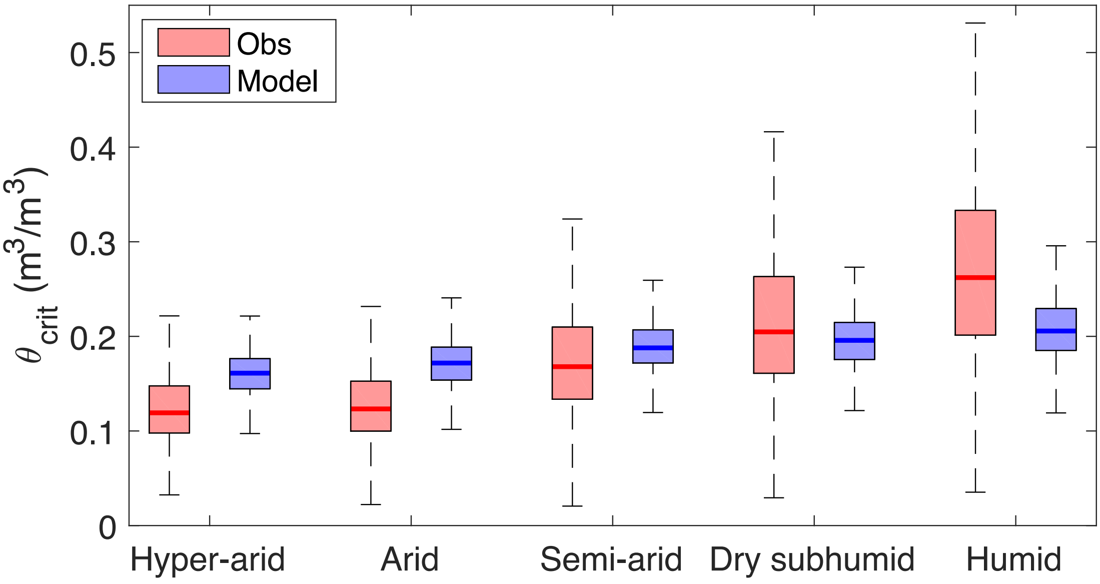

Supplementary Fig. 11. Comparison between multi-model mean critical soil moisture threshold (θ_{crit}) and observation-based θ_{crit} grouped by climate types based on the aridity classification. For each box plot, the middle line indicates the median; the box indicates the upper and lower quartiles, and the whiskers indicate the 5th and 95th percentiles of the data. Obs: observations.

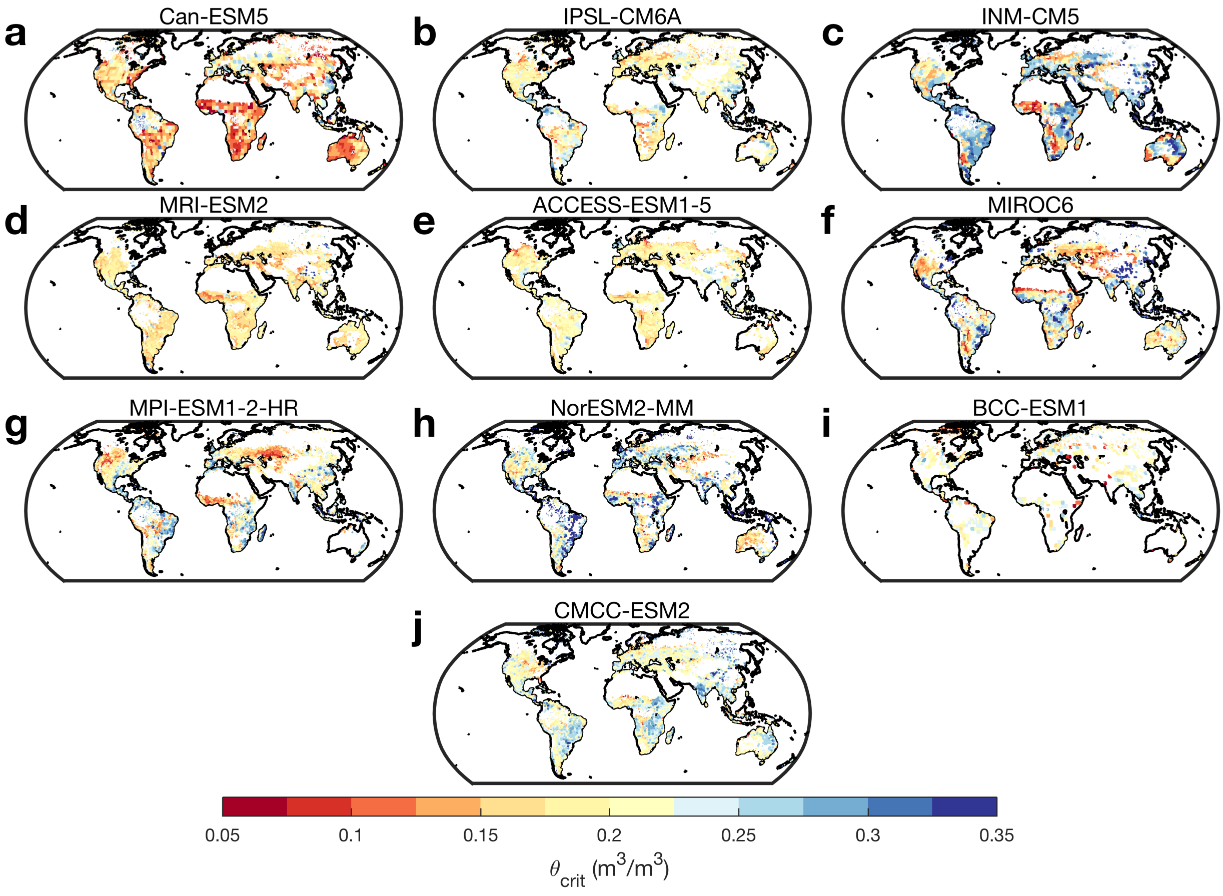

Supplementary Fig. 12. The critical soil moisture threshold (θ_{crit}) from ten Earth System Models with daily outputs.

Supplementary Table 1. Eddy covariance sites used in this study. Site identifier (ID), latitude (Lat, °), longitude (Long, °), plant functional type (PFT), and study periods are listed. Plant functional types were defined according to the IGBP classification, including SAV (savannas); SHR (shrublands); ENF (evergreen needleleaf forests); EBF (evergreen broadleaf forests); DBF (deciduous broadleaf forests); MF (mixed forests); GRA (grasslands) and CRO (croplands).

Site ID	Lat	Long	IGBP	Periods	Reference
AU-Ade	-13.08	131.12	WSA	2007-2009	[5]
AU-ASM	-22.28	133.25	SAV	2010-2014	[6]
AU-DaP	-14.06	131.32	GRA	2007-2013	[7]
AU-DaS	-14.16	131.39	SAV	2008-2014	[8]
AU-Gin	-31.38	115.71	SAV	2011-2014	[9]
AU-Rig	-36.65	145.58	GRA	2011-2014	[10]
AU-Whr	-36.67	145.03	EBF	2011-2014	[11]
CA-Oas	53.63	-106.20	DBF	1996-2010	[12]
CH-Cha	47.21	8.41	GRA	2005-2020	[13]
CH-Dav	46.82	9.86	ENF	1997-2014	[14]
CH-Oe1	47.29	7.73	GRA	2002-2008	[15]
CZ-BK2	49.49	18.54	GRA	2004-2012	[16]
CZ-Lnz	48.68	16.95	MF	2015-2020	[17]
DE-Gri	50.95	13.51	GRA	2004-2020	[18]
DE-Hai	51.08	10.45	DBF	2000-2020	[19]
DE-HoH	52.09	11.22	DBF	2015-2020	[20]
DE-Kli	50.89	13.52	CRO	2004-2018	[21]
DE-Lnf	51.33	10.37	DBF	2002-2012	[22]
DE-Obe	50.79	13.72	ENF	2008-2020	[23]
DE-RuR	50.62	6.30	GRA	2011-2020	[24]
DE-RuS	50.87	6.45	CRO	2011-2021	[25]
DE-Tha	50.96	13.57	ENF	1996-2020	[26]
ES-Abr	38.70	-6.79	SAV	2015-2018	[27]
ES-LM1	39.94	-5.78	SAV	2014-2020	[27]
ES-LM2	39.93	-5.78	SAV	2014-2020	[27]
FR-Hes	48.67	7.06	DBF	2014-2020	[28]
GF-Guy	5.28	-52.92	EBF	2004-2014	[29]
IT-CA2	42.38	12.03	CRO	2011-2014	[30]
IT-Col	41.85	13.59	DBF	1996-2014	[31]
IT-Lsn	45.74	12.75	SHR	2016-2020	[20]
IT-Noe	40.61	8.15	SHR	2004-2014	[32]
IT-SR2	43.73	10.29	ENF	2013-2020	[17]
IT-SRo	43.73	10.28	ENF	1999-2012	[33]
JP-MBF	44.39	142.32	DBF	2003-2005	[34]
NL-Loo	52.17	5.74	ENF	1996-2018	[35]
SE-Htm	56.10	13.42	ENF	2015-2020	[20]

US-AR1	36.43	-99.42	GRA	2009-2012	[36]
US-Goo	34.25	-89.87	GRA	2002-2006	[37]
US-Me3	44.32	-121.61	ENF	2004-2009	[38]
US-MMS	39.32	-86.41	DBF	1999-2014	[39]
US-SRM	31.82	-110.87	SAV	2004-2014	[40]
US-Var	38.41	-120.95	GRA	2000-2014	[41]
US-WCr	45.81	-90.08	DBF	1999-2014	[42]
US-Whs	31.74	-110.05	SHR	2007-2014	[43]

Supplementary Table 2. The predictor variables used in the random forest models.

Variable	Variable group	Units	Sources
Sand	Soil	%	ref[44] (SoilGrids: https://www.isric.org/explore/soilgrids)
Silt	Soil	%	
Clay	Soil	%	
Soil organic carbon	Soil	g/kg	
Organic carbon stock	Soil	kg/m³	
Organic carbon density	Soil	kg/m³	
Total Nitrogen	Soil	g/kg	
Coarse fragments	Soil	%	
Cation exchange capacity at pH7	Soil	cmol/kg	
Bulk density	Soil	kg/dm³	
pH	Soil	Unitless	
Total Phosphorus	Soil	g/kg	ref[45]
Aridity index	Climatic	Unitless	ref[46] (Global-AI_PET_v3)
Potential evapotranspiration (PET)	Climatic	mm	
Precipitation frequency	Climatic	days	ERA5-Land (https://cds.climate.copernicus.eu/)
Vapour pressure deficit	Climatic	kPa	ref[47]
Radiation	Climatic	W/m^2	
Wind speed	Climatic	m/s	
Mean annual precipitation	Climatic	mm	
Albedo	Climatic	Unitless	MODIS (https://lpdaac.usgs.gov/products/mcd43c3v061/)
Leaf area index	Vegetative	m^2/m^2	ref[48]
EVI	Vegetative	EVI value	ref[49] (MODIS)
NDVI	Vegetative	NDVI value	
Tree cover	Vegetative	%	ref[50]
Tree density	Vegetative	Number of trees/ha	ref[51]

Woody density	Vegetative	mg/mm^3	ref[52]
Forest canopy height	Vegetative	m	ref[53]
Root depth	Vegetative	m	ref[54]
Specific leaf area	Vegetative	m^2/kg	ref[55]
Leaf Nitrogen	Vegetative	mg/g	
Leaf Phosphorus	Vegetative	mg/g	
Plant hydraulic resistance	Plant hydraulic traits	day/mm	ref[54]
Leaf water potential at 50% of xylem conductance (P50)	Plant hydraulic traits	MPa	ref[56]
The slope parameter in the Medlyn's stomatal conductance model	Plant hydraulic traits	kPa/0.5	
Maximum xylem conductance	Plant hydraulic traits	mm/hr/MPa	
The ratio between the leaf water potential at 50% of stomatal conductance and that at 50% of xylem conductance	Plant hydraulic traits	Unitless	

Supplementary Table 3. Earth System Models from CMIP6 models with daily outputs used in the analysis.

CMIP6 Models	Institution ID	Modeling Group	Land Component	Reference
ACCESS-ESM1-5	CSIRO	Commonwealth Scientific and Industrial Research Organisation, Australia	CABLE2.4	57
BCC-ESM1	BCC	Beijing Climate Center	BCC_AVIM2	58
Can-ESM5	CCCma	Canadian Centre for Climate Modelling and Analysis	CLASS3.6/CTEM1.2	59
CMCC-ESM2	CMCC	Fondazione Centro Euro-Mediterraneo sui Cambiamenti Climatici, Italy	CLM4.5 (BGC mode)	60
INM-CM5-0	INM	Institute for Numerical Mathematics	INM-LND1	61
IPSL-CM6A-LR	IPSL	Institute Pierre Simon Laplace, France	ORCHIDEE (v2.0)	62
MIROC6	MIROC	Japan Agency for Marine-Earth Science and Technology	MATSIRO6.0	63
MPI-ESM1-2-HR	MPI-M	Max Planck Institute for Meteorology	JSBACH3.20	64
MRI-ESM2-0	MRI	Meteorological Research Institute, Japan	HAL 1.0	65
NorESM2-MM	NCC	Norwegian Climate Centre, Norway	CLM	66

Supplementary References

1. Monfreda C, Ramankutty N, Foley JA. Farming the planet: 2. Geographic distribution of crop areas, yields, physiological types, and net primary production in the year 2000. *Global biogeochemical cycles* **22**, (2008).

2. Siebert S, Henrich V, Frenken K, Burke J. Global map of irrigation areas version 5. *Rheinische Friedrich-Wilhelms-University, Bonn, Germany/Food and Agriculture Organization of the United Nations, Rome, Italy* **2**, 1299-1327 (2013).

3. Potapov P*, et al.* Global maps of cropland extent and change show accelerated cropland expansion in the twenty-first century. *Nature Food* **3**, 19-28 (2022).

4. Middleton N, Thomas D. *World atlas of desertification.. ed. 2*. Arnold, Hodder Headline, PLC (1997).

5. Beringer J*, et al.* SPECIAL—Savanna patterns of energy and carbon integrated across the landscape. *Bulletin of the American Meteorological Society* **92**, 1467-1485 (2011).

6. Barraza V*, et al.* Estimation of latent heat flux over savannah vegetation across the North Australian Tropical Transect from multiple sensors and global meteorological data. *Agricultural and Forest Meteorology* **232**, 689-703 (2017).

7. Zhuang W*, et al.* How energy and water availability constrain vegetation water-use along the North Australian Tropical Transect. *International Journal of Plant Production* **10**, (2016).

8. Cernusak LA, Hutley LB, Beringer J, Holtum JA, Turner BL. Photosynthetic physiology of eucalypts along a sub-continental rainfall gradient in northern Australia. *Agricultural and Forest Meteorology* **151**, 1462-1470 (2011).

9. Macfarlane C, Lambert P, Byrne J, Johnstone C, Smart N. FLUXNET2015 AU-Gin Gingin. (ed^(eds). FluxNet; Edith Cowan University (Centre for Ecosystem Management) (2016).

10. Azmi M, Rüdiger C, Walker JP. Statistical analysis of short-term water stress conditions at Riggs Creek OzFlux tower site. *Theoretical and Applied Climatology*, 1-13 (2016).

11. van Gorsel E*, et al.* Carbon uptake and water use in woodlands and forests in southern Australia during an extreme heat wave event in the "Angry Summer" of 2012/2013. *Biogeosciences, 2016, vol 13, núm 21, p 5947-5964*, (2016).

12. Barr AG, Black T, Hogg E, Kljun N, Morgenstern K, Nesic Z. Inter-annual variability in the leaf area index of a boreal aspen-hazelnut forest in relation to net ecosystem production. *Agricultural and forest meteorology* **126**, 237-255 (2004).

13. Zeeman MJ, *et al.* Management and climate impacts on net CO 2 fluxes and carbon budgets of three grasslands along an elevational gradient in Switzerland. *Agricultural and Forest Meteorology* **150**, 519-530 (2010).

14. Zielis S, Etzold S, Zweifel R, Eugster W, Haeni M, Buchmann N. NEP of a Swiss subalpine forest is significantly driven not only by current but also by previous year's weather. *Biogeosciences* **11**, 1627-1635 (2014).

15. Ammann C, Flechard CR, Leifeld J, Neftel A, Fuhrer J. The carbon budget of newly established temperate grassland depends on management intensity. *Agr Ecosyst Environ* **121**, 5-20 (2007).

16. Sigut L, *et al.* FLUXNET2015 CZ-BK2 Bily Kriz grassland. (ed^(eds). FluxNet; Global Change Research Institute CAS (2016).

17. Gourlez de la Motte L, *et al.* Non-stomatal processes reduce gross primary productivity in temperate forest ecosystems during severe edaphic drought. *Philosophical Transactions of the Royal Society B* **375**, 20190527 (2020).

18. Hussain M, *et al.* Summer drought influence on CO 2 and water fluxes of extensively managed grassland in Germany. *Agriculture, ecosystems & environment* **141**, 67-76 (2011).

19. Ahrends HE, *et al.* Tree phenology and carbon dioxide fluxes: use of digital photography for process-based interpretation at the ecosystem scale. *Climate Research* **39**, 261-274 (2009).

20. Warm Winter 2020 Team, & ICOS Ecosystem Thematic Centre. Warm Winter 2020 ecosystem eddy covariance flux product for 73 stations in FLUXNET-Archive format— release 2022-1 (version 1.0). ICOS Carbon Portal; https://doi.org/10.18160/2G60-ZHAK. (2022).

21. Prescher A-K, Grünwald T, Bernhofer C. Land use regulates carbon budgets in eastern Germany: From NEE to NBP. *Agricultural and Forest Meteorology* **150**, 1016-1025 (2010).

22. Anthoni P, *et al.* Forest and agricultural land-use-dependent CO2 exchange in Thuringia, Germany. *Global Change Biology* **10**, 2005-2019 (2004).

23. Zimmermann F, Plessow K, Queck R, Bernhofer C, Matschullat J. Atmospheric N-and S-fluxes to a spruce forest—Comparison of inferential modelling and the throughfall method. *Atmospheric Environment* **40**, 4782-4796 (2006).

24. Post H, Hendricks Franssen H-J, Graf A, Schmidt M, Vereecken H. Uncertainty analysis of eddy covariance CO 2 flux measurements for different EC tower distances using an extended two-tower approach. *Biogeosciences* **12**, 1205-1221 (2015).

25. Mauder M, *et al.* A strategy for quality and uncertainty assessment of long-term eddy-covariance measurements. *Agricultural and Forest Meteorology* **169**, 122-135 (2013).

26. Grünwald T, Bernhofer C. A decade of carbon, water and energy flux measurements of an old spruce forest at the Anchor Station Tharandt. *Tellus B* **59**, 387-396 (2007).

27. El-Madany TS, *et al.* Drought and heatwave impacts on semi-arid ecosystems' carbon fluxes along a precipitation gradient. *Philosophical Transactions of the Royal Society B* **375**, 20190519 (2020).

28. Granier A, *et al.* The carbon balance of a young Beech forest. *Funct Ecol* **14**, 312-325 (2000).

29. Bonal D, *et al.* Impact of severe dry season on net ecosystem exchange in the Neotropical rainforest of French Guiana. *Global Change Biology* **14**, 1917-1933 (2008).

30. Sabbatini S, *et al.* Greenhouse gas balance of cropland conversion to bioenergy poplar short rotation coppice. *Biogeosciences Discussions* **12**, (2015).

31. Valentini R, Angelis Pd, Matteucci G, Monaco R, Dore S, Mucnozza GS. Seasonal net carbon dioxide exchange of a beech forest with the atmosphere. *Global Change Biology* **2**, 199-207 (1996).

32. Reichstein M, *et al.* Inverse modeling of seasonal drought effects on canopy CO2/H2O exchange in three Mediterranean ecosystems. *Journal of Geophysical Research: Atmospheres* **108**, (2003).

33. Chiesi M, *et al.* Modelling carbon budget of Mediterranean forests using ground and remote sensing measurements. *Agricultural and Forest Meteorology* **135**, 22-34 (2005).

34. Matsumoto K, *et al.* Energy consumption and evapotranspiration at several boreal and temperate forests in the Far East. *Agricultural and Forest Meteorology* **148**, 1978-1989 (2008).

35. Gioli B, *et al.* Comparison between tower and aircraft-based eddy covariance fluxes in five European regions. *Agricultural and Forest Meteorology* **127**, 1-16 (2004).

36. Holmes TR, Hain CR, Anderson MC, Crow WT. Cloud tolerance of remote-sensing technologies to measure land surface temperature. *Hydrology and Earth System Sciences* **20**, 3263 (2016).

37. Runkle BR, *et al.* Delta-flux: An eddy covariance network for a climate-smart lower Mississippi Basin. *Agricultural & Environmental Letters* **2**, ael2017.2001.0003 (2017).

38. Barr A*, et al.* Use of change-point detection for friction–velocity threshold evaluation in eddy-covariance studies. *Agricultural and forest meteorology* **171**, 31-45 (2013).

39. Schmid HP, Grimmond CSB, Cropley F, Offerle B, Su H-B. Measurements of CO 2 and energy fluxes over a mixed hardwood forest in the mid-western United States. *Agricultural and Forest Meteorology* **103**, 357-374 (2000).

40. Scott RL, Jenerette GD, Potts DL, Huxman TE. Effects of seasonal drought on net carbon dioxide exchange from a woody-plant-encroached semiarid grassland. *Journal of Geophysical Research: Biogeosciences* **114**, (2009).

41. Ma S, Baldocchi DD, Xu L, Hehn T. Inter-annual variability in carbon dioxide exchange of an oak/grass savanna and open grassland in California. *Agricultural and Forest Meteorology* **147**, 157-171 (2007).

42. Cook BD*, et al.* Carbon exchange and venting anomalies in an upland deciduous forest in northern Wisconsin, USA. *Agricultural and Forest Meteorology* **126**, 271-295 (2004).

43. Scott RL, Biederman JA, Hamerlynck EP, Barron-Gafford GA. The carbon balance pivot point of southwestern US semiarid ecosystems: Insights from the 21st century drought. *Journal of Geophysical Research: Biogeosciences* **120**, 2612-2624 (2015).

44. Poggio L*, et al.* SoilGrids 2.0: producing soil information for the globe with quantified spatial uncertainty. *Soil* **7**, 217-240 (2021).

45. He X*, et al.* Global patterns and drivers of soil total phosphorus concentration. *Earth System Science Data* **13**, 5831-5846 (2021).

46. Zomer RJ, Xu J, Trabucco A. Version 3 of the Global Aridity Index and Potential Evapotranspiration Database. *Scientific Data* **9**, 1-15 (2022).

47. Abatzoglou JT, Dobrowski SZ, Parks SA, Hegewisch KC. TerraClimate, a high-resolution global dataset of monthly climate and climatic water balance from 1958–2015. *Scientific data* **5**, 1-12 (2018).

48. Liu Y, Liu R, Chen JM. Retrospective retrieval of long-term consistent global leaf area index (1981–2011) from combined AVHRR and MODIS data. *Journal of Geophysical Research: Biogeosciences* **117**, (2012).

49. Didan K. MOD13Q1 MODIS/Terra Vegetation Indices 16-Day L3 Global 250m SIN Grid V006. NASA EOSDIS LP DAAC. *Retrieved from doi* **10**, (2015).

50. Hansen MC, *et al.* High-resolution global maps of 21st-century forest cover change. *Science* **342**, 850-853 (2013).

51. Crowther TW, *et al.* Mapping tree density at a global scale. *Nature* **525**, 201-205 (2015).

52. Boonman CC, *et al.* Assessing the reliability of predicted plant trait distributions at the global scale. *Global ecology and biogeography* **29**, 1034-1051 (2020).

53. Simard M, Pinto N, Fisher JB, Baccini A. Mapping forest canopy height globally with spaceborne lidar. *Journal of Geophysical Research: Biogeosciences* **116**, (2011).

54. Liu Y, Konings AG, Kennedy D, Gentine P. Global coordination in plant physiological and rooting strategies in response to water stress. *Global Biogeochemical Cycles* **35**, e2020GB006758 (2021).

55. Butler EE, *et al.* Mapping local and global variability in plant trait distributions. *Proceedings of the National Academy of Sciences* **114**, E10937-E10946 (2017).

56. Liu Y, Holtzman NM, Konings AG. Global ecosystem-scale plant hydraulic traits retrieved using model–data fusion. *Hydrology and Earth System Sciences* **25**, 2399-2417 (2021).

57. Ziehn T, *et al.* The Australian Earth System Model: ACCESS-ESM1. 5. *Journal of Southern Hemisphere Earth Systems Science*, (2020).

58. Zhang J, *et al.* BCC-ESM1 Model Datasets for the CMIP6 Aerosol Chemistry Model Intercomparison Project (AerChemMIP). *Advances in Atmospheric Sciences* **38**, 317-328 (2021).

59. Swart NC, *et al.* The Canadian earth system model version 5 (CanESM5. 0.3). *Geoscientific Model Development* **12**, 4823-4873 (2019).

60. Cherchi A, *et al.* Global Mean Climate and Main Patterns of Variability in the CMCC-CM2 Coupled Model. *Journal of Advances in Modeling Earth Systems* **11**, 185-209 (2019).

61. Volodin E. Possible Climate Change in Russia in the 21st Century Based on the INM-CM5-0 Climate Model. *Russian Meteorology and Hydrology* **47**, 327-333 (2022).

62. Lurton T, *et al.* Implementation of the CMIP6 Forcing Data in the IPSL-CM6A-LR Model. *Journal of Advances in Modeling Earth Systems* **12**, e2019MS001940 (2020).

63. Tatebe H*, et al.* Description and basic evaluation of simulated mean state, internal variability, and climate sensitivity in MIROC6. *Geoscientific Model Development* **12**, 2727-2765 (2019).

64. Gutjahr O*, et al.* Max planck institute earth system model (MPI-ESM1. 2) for the high-resolution model intercomparison project (HighResMIP). *Geoscientific Model Development* **12**, 3241-3281 (2019).

65. Yukimoto S*, et al.* The Meteorological Research Institute Earth System Model version 2.0, MRI-ESM2. 0: Description and basic evaluation of the physical component. *Journal of the Meteorological Society of Japan Ser II*, (2019).

66. Seland Ø*, et al.* The Norwegian Earth System Model, NorESM2–Evaluation of theCMIP6 DECK and historical simulations. *Geoscientific Model Development Discussions*, 1-68 (2020).
